# Transfer of Knowledge and Academic-Practice Partnership in Nursing Homes - International Experiences

**DOI:** 10.1093/geroni/igaf122.324

**Published:** 2025-12-31

**Authors:** Karin Wolf-Ostermann, Hilde Verbeek, Marie Boltz

## Abstract

Nursing homes are not only places where people in need of care are cared for, they are also increasingly seen as places of partnership between academia and practice and places of knowledge transfer. They offer practical approaches to skills development and lifelong learning for staff, promote research capacity and improve outcomes for residents. This requires continuous organizational development, the expansion of digitalization potential, the training of specialized geriatric staff and the integration of evidence-based, person-centred care into the operation of nursing homes. Academic-Practice Partnership models are therefore gaining increasingly attention as a feasible approach to achieve these goals. This symposium will describe interdisciplinary collaboration between scientists, care providers and educators in nursing homes in four countries: United States, Germany, the Netherlands, and the Austria. Experiences of stakeholders, research activities and the evaluation and implementation of digital services will be discussed. The first presentation will presentation will provide an overview of the Dutch Living Lab in Long-term care. The second presentation will describe interdisciplinary staff’s experiences and the impact of Age-Friendly Care 4M Rounding with older adults living in long-term care. The third presentation will highlight the collaborative development and implementation of a practice-oriented, evidence-based toolkit for non-pharmacological pain management within the nursing home environment of the Austrian living lab. The final presentation will describe the TCALL project in Germany, with a focus on implementing innovative digital tools into daily practice. A discussion will follow addressing future directions and challenges of academic-practice partnership in nursing homes including policy implications.

